# Engineering glycoside hydrolase stability by the introduction of zinc binding

**DOI:** 10.1107/S2059798318006678

**Published:** 2018-06-27

**Authors:** Thomas L. Ellinghaus, Jose H. Pereira, Ryan P. McAndrew, Ditte H. Welner, Andy M. DeGiovanni, Joel M. Guenther, Huu M. Tran, Taya Feldman, Blake A. Simmons, Kenneth L. Sale, Paul D. Adams

**Affiliations:** a Joint BioEnergy Institute, Emeryville, CA 94608, USA; b Molecular Biophysics and Integrated Bioimaging Division, Lawrence Berkeley National Laboratory, Berkeley, CA 94720, USA; c Biological and Engineering Sciences Center, Sandia National Laboratories, Livermore, CA 94551, USA; d Biological Systems and Engineering Division, Lawrence Berkeley National Laboratory, Berkeley, CA 94720, USA; e Department of Bioengineering, University of California, Berkeley, CA 94720, USA

**Keywords:** glycoside hydrolases, protein engineering, thermal stability, X-ray crystallography

## Abstract

The development of robust enzymes, in particular cellulases, is a key step in the success of biological routes to ‘second-generation’ biofuels. The typical sources of the enzymes used to degrade biomass include mesophilic and thermophilic organisms. The endoglucanase J30 from glycoside hydrolase family 9 was originally identified through metagenomic analyses of compost-derived bacterial consortia. These studies, which were tailored to favor growth on targeted feedstocks, have already been shown to identify cellulases with considerable thermal tolerance. The amino-acid sequence of J30 shows comparably low identity to those of previously analyzed enzymes. As an enzyme that combines a well measurable activity with a relatively low optimal temperature (50°C) and a modest thermal tolerance, it offers the potential for structural optimization aimed at increased stability. Here, the crystal structure of wild-type J30 is presented along with that of a designed triple-mutant variant with improved characteristics for industrial applications. Through the introduction of a structural Zn^2+^ site, the thermal tolerance was increased by more than 10°C and was paralleled by an increase in the catalytic optimum temperature by more than 5°C.

## Abstract 2

The engineering of metal binding into a cellulase increases its temperature stability while maintaining its other catalytic properties.

## 1. Introduction   

Concerns about climate, increasing energy demands and the limited long-term supply of fossil fuels, as well as ethical considerations, have promoted research on more sustainable lignocellulosic ‘second-generation’ biofuels. They rely on the deconstruction of cellulose and hemicellulose from feedstocks such as switchgrass into monosaccharides. One step in this process, saccharification, employs mixtures of thermally and chemically robust glycoside hydrolases (GHs). These mixtures typically make use of the combined activities of endoglucanases (EC 3.2.1.4), β-glucosidases (EC 3.2.1.21) and cellobiohydrolases (EC 3.2.1.91 and EC 3.2.1.176; CBHs). The Carbohydrate-Active enZymes database (CAZy; http://www.cazy.org) currently lists 153 different GH families based on amino-acid sequence similarities. This classification facilitates the grouping of GHs according to structural, mechanistic and evolutionary aspects independent of substrate specificities.

J30, an endoglucanase with activity against *p*-nitrophenyl cellobiose (*p*NPC), which produces cellobiose as its primary product, was identified and analyzed from metagenomic data originating from a switchgrass-adapted microbial community (Allgaier *et al.*, 2010 ▸; D’haeseleer *et al.*, 2013 ▸; Gladden *et al.*, 2014 ▸). Its likely source is the moderately thermophilic bacterium *Thermobacillus composti* KWC4, and its catalytic optimum is 50°C. It is annotated as a GH family 9 member consisting of an N-terminal Ig-like domain and a C-terminal catalytic domain. Initial annotations, based on dbCAN, suggested the presence of a carbohydrate-binding module (CBM; for example, a CBM family 30 motif) within the Ig-like domain. However, the more recent dbCAN2 meta server (http://cys.bios.niu.edu/dbCAN2) does not identify a CBM. With 54% sequence identity, an uncharacterized family 9 GH from *T. composti* is the closest homolog of J30. Of those with solved structures, the leaf-branch compost CelG (LC-CelG; PDB entry 3x17; Okano *et al.*, 2015 ▸) is most similar with 41% identity, followed by Cel9A from *Alicyclobacillus acido­caldarius* (AaCel9A; PDB entry 3ez8; Pereira *et al.*, 2009 ▸) and Cel9A from *Clostridium thermocellum* (CtCel9A; PDB entry 1clc; M. B. Lascombe, H. Souchon, M. Juy & P. M. Alzari, unpublished work) with 32 and 31% identity, respectively. Among the less similar family members found in the Protein Data Bank (Berman *et al.*, 2000 ▸) are crystal structures without an Ig-like domain and those carrying a C-terminally adjacent family 3 CBM, as summarized in greater detail elsewhere (Okano *et al.*, 2015 ▸).

We have solved the structure of J30 to 1.7 Å resolution. The enzyme represents one of only three structures of a GH family 9 member free of structural Ca^2+^ and Zn^2+^ ions, the others being a chitobiase from *Vibrio parahaemolyticus* (PDB entry 3h7l; New York SGX Research Center for Structural Genomics, unpublished work) and a glucosaminidase from *Photobacterium profundum* (PpGlcNase; PDB entry 5dgq; Honda *et al.*, 2016 ▸). Among the other members with solved structures, those exhibiting a considerably higher thermal tolerance than J30 contain one to four structural metal ions (Schubot *et al.*, 2004 ▸; Kesavulu *et al.*, 2012 ▸; Okano *et al.*, 2015 ▸; Eckert *et al.*, 2002 ▸; Brunecky *et al.*, 2013 ▸; Chauvaux *et al.*, 1995 ▸). In light of the stabilization of CtCel9A through metal ion binding (Chauvaux *et al.*, 1995 ▸, 1990 ▸), we hypothesized that the introduction of a Zn^2+^ ion could improve the thermal stability of J30. Using the CtCel9A crystal structure as a reference model and comparing the geometry of its Zn^2+^-binding site with the corresponding segments within J30, we created a mutant called J30 CCH (denoting the residue identities of the mutations: A98**C**, G114**C** and Y143**H**). The crystal structure of the triple mutant showed the successful introduction of a Zn^2+^ ion at the intended binding site. Our circular-dichroism (CD) and differential scanning fluorimetry (DSF) data demonstrate a successful increase in the melting temperature of J30. An enzymatic analysis of the enzyme variants showed an increase in the catalytic optimum temperature by 5–10°C. Our data support the proposed role of structural metal ions in promoting thermal stability (Juy *et al.*, 1992 ▸) and underline the suitability of metal introduction as a tool to engineer robust proteins in the context of biomass saccharification and beyond (Browner *et al.*, 1994 ▸; Plegaria *et al.*, 2015 ▸).

## 2. Methods   

### 2.1. Molecular biology, protein expression and purification   

The A98C, G114C and Y143H mutations were introduced at the DNA level into plasmid pBIL-J30 using the QuikChange Lightning Multi Site-Directed Mutagenesis Kit (Agilent Technologies, catalog No. 210515).

Wild-type J30 (J30 wt) and J30 CCH, each carrying a C-terminal polyhistidine tag, were produced in *Escherichia coli* NEB Express cells grown in TB medium with 50 µg ml^−1^ kanamycin and 2 m*M* MgSO_4_ at 200 rev min^−1^. The bacterial suspensions were incubated at 37°C before induction with 0.5 m*M* IPTG at an OD_600 nm_ of 4 (J30 wt) or 1 (J30 CCH) and at 20°C thereafter. The protein expressions lasted 17 h (J30 wt) or 19 h (J30 CCH) until an OD_600 nm_ of 20 (J30 wt) or 20.5 (J30 CCH) was attained.

The cells were lysed in a buffer consisting of 0.15 *M* NaCl, 25 m*M* HEPES pH 7.4 with 1 m*M* PMSF using an Avestin EmulsiFlex-C3 homogenizer. The proteins were purified using an ÄKTAexplorer 100 Air by nickel-affinity (5 ml HisTrap HP or FF) and size-exclusion chromatography (Superdex 200 10/300 GL, all from GE Healthcare Life Sciences). All of the media and buffers used to produce and isolate J30 CCH also contained 10 µ*M* ZnCl_2_.

### 2.2. Crystallization   

Initial sparse-matrix crystallization screening of wild-type J30 was conducted using the following screens: Berkeley (from Lawrence Berkeley National Laboratory; Pereira *et al.*, 2017 ▸), Crystal Screen, Index, Natrix, PEG/Ion, PEGRx, SaltRx (all from Hampton Research) and MCSG-1 (from Microlytic) (Jancarik & Kim, 1991 ▸). They were set up using a Phoenix Robot (Art Robbins Instruments). At a concentration of 12 mg ml^−1^ purified enzyme, the protein was crystallized in 0.1 *M* trisodium citrate dihydrate pH 5, 15%(*w*/*v*) 2-propanol, 10%(*w*/*v*) PEG 10 000. Suitably sized crystals were obtained after 3 d of growth at room temperature using the sitting-drop vapor-diffusion method. J30 CCH crystals were prepared in the same manner. All drops consisted of 1 µl protein solution and 0.5 µl reservoir solution.

### 2.3. Data collection and processing   

The crystals were transferred through 20%(*v*/*v*) glycerol and flash-cooled in liquid nitrogen. Native J30 data sets were collected on beamline 7-1 at the Stanford Synchrotron Radiation Lightsource (SSRL), SLAC National Accelerator Laboratory, and the J30 CCH diffraction data were recorded on the Berkeley Center for Structural Biology beamline 5.0.1 of the Advanced Light Source (ALS) at Lawrence Berkeley National Laboratory. All data were measured using an oscillation angle Δφ of 1° and ADSC Quantum 315r detectors.

All data sets were processed using the -3dii option in *xia*2 (Winter, 2010 ▸; Kabsch, 2010 ▸; Evans, 2006 ▸). Phasing was carried out by molecular replacement using *Phaser* (McCoy *et al.*, 2007 ▸) from the *PHENIX* suite of programs (Adams *et al.*, 2010 ▸). CtCel9A (PDB entry 1clc; 31% sequence identity) served as a search model for J30 wt (top TFZ score of 19.7), which in turn was used to generate the J30 CCH maps. Model improvement employed alternating rounds of manual modelling using *Coot* (Emsley *et al.*, 2010 ▸) and automated structure refinement by *phenix.refine* (Afonine *et al.*, 2012 ▸). 2000 reflections in each data set were randomly selected for cross-validation. Later iterations included TLS refinement using TLS groups obtained from the *TLSMD* web server (Painter & Merritt, 2006*a* ▸,*b* ▸). All data-collection, phasing and refinement statistics are summarized in Table 1 ▸. The figures showing structural models were created using *PyMOL* (v.1.7.2.1; Schrödinger).

### 2.4. Differential scanning fluorimetry   

DSF assays comparing the thermal stability of J30 wt and J30 CCH were performed on an Applied Biosciences StepOnePlus Real-Time PCR System in triplicate on a 30 µl scale containing 0.5 mg ml^−1^ enzyme, 150 m*M* NaCl, 25 m*M* HEPES pH 7.4, 10 µ*M* ZnCl_2_ and SYPRO Orange Protein Gel Stain (Life Technologies, catalog No. S-6650) at 5×. Fluorescence signals were recorded from 25 to 99°C.

The Zn^2+^-titration samples were prepared by a 1 h incubation of J30 CCH with a 150-fold stoichiometric excess of EDTA pH 7.4 and dialysis for 18 h against 0.15 *M* NaCl, 25 m*M* HEPES buffer pH 7.4. A 1 mg ml^−1^ enzyme solution was incubated with ZnCl_2_ at various concentrations for 30 min before the addition of the dye. The DSF assays were carried out as above and at final concentrations of 0.5 mg ml^−1^ J30 CCH, 0–10 µ*M* ZnCl_2_, 0.15 *M* NaCl, 25 m*M* HEPES pH 7.4 with SYPRO Orange Protein Gel Stain at 5×.

### 2.5. Circular dichroism   

Melting curves of J30 wt and J30 CCH from *E. coli* were recorded from 20 to 85°C with a Jasco J-815 CD spectrometer using 1 mm cuvettes. The assay concentrations were 0.1 mg ml^−1^ enzyme, 150 m*M* NaCl, 25 m*M* HEPES pH 7.4, 10 µ*M* ZnCl_2_. Three accumulations were recorded in the wavelength range 200–250 nm applying 0.2 nm data pitches.

### 2.6. Enzyme-specificity assays   

Reactions were set up on a 50 µl scale using 15 µg ml^−1^ enzyme, 0.1 *M* MES pH 6, 10 µ*M* ZnCl_2_, and 0.25 m*M* 4-nitrophenyl β-d-glucopyranoside (*p*NPG; Sigma, catalog No. N7006), 4-nitrophenyl β-d-cellobioside (*p*NPC; TCI America, catalog No. N0867) or 4-nitrophenyl β-d-cellotrioside (*p*NPG3; Carbosynth, catalog No. EN04796). Positive controls contained a final concentration of 3.6 *M* NaOH instead of enzyme. Negative controls contained buffer instead of enzyme, and were subtracted before internal standardization against the alkaline hydrolysis. Triplicate samples were incubated at 50°C in Applied Biosciences Veriti 96-Well Thermal Cyclers, cooled to 4°C, transferred to 96-well clear flat-bottom plates and mixed with 2%(*w*/*v*) sodium carbonate in a 1:1 volume ratio. The absorbance at an incident wavelength of 405 nm was measured on a Molecular Devices SpectraMax M2.

### 2.7. Catalytic profiling   

The assay conditions comprised 15 µg ml^−1^ enzyme, 0.3 m*M* *p*NPC (Sigma, catalog No. N5759), 10 µ*M* ZnCl_2_, 31.3 m*M* citrate and, depending on the pH, 4.7–51.1 m*M* lactate and 28.4–59.4 m*M* phosphate. 100 µl reactions were set up in triplicate in 96-well plates on BioExpress GeneMate IsoFreeze PCR racks and incubated for 30 min in Applied Biosciences Veriti 96-Well Thermal Cyclers. The sample-cooling and transfer, reaction-quenching and readout steps were carried out as described above, except that Beckman Coulter Biomek FX and NX^P^ robots were used for all of the liquid handling in the 96-well format plates. Negative controls contained buffer instead of enzyme and were subtracted.

## 3. Results   

### 3.1. Overall fold of J30   

**Figure 1:**
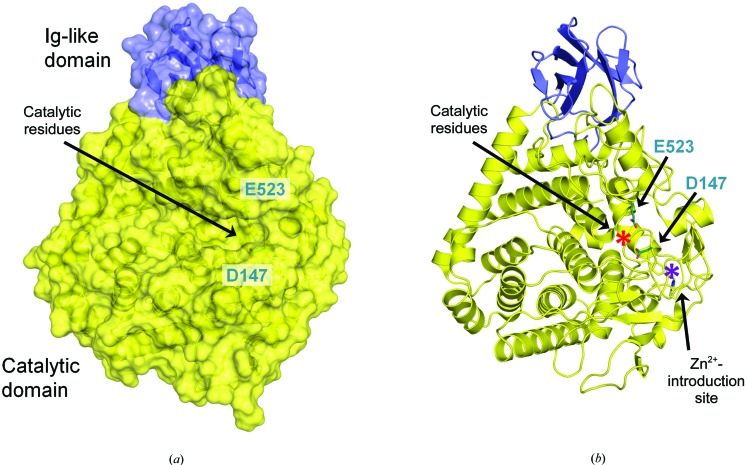
Crystal structure of J30 as a cartoon representation with (*a*) and without (*b*) the protein surface. J30 wt contains an N-terminal Ig-like domain and a C-­terminal catalytic domain, as is common among GH family 9 members. The side chains of the catalytic amino-acid residues Asp147 and Glu523 are depicted in teal. Also highlighted are the side chains corresponding to the residues mediating Zn^2+^ coordination in the triple mutant J30 CCH. (*a*) Residues Asp147 and Glu523 are positioned at the active site and in the center of the substrate-binding channel. (*b*) The active site (red asterisk) and Zn^2+^-introduction site (purple asterisk) are separate sites in proximity to one another. Within the catalytic domain, the (α/α)_6_-barrel structure (bottom left) and the four-stranded β-sheet (bottom right) are turned into perspective.

The crystal structure of J30 was solved to a resolution of 1.7 Å by molecular replacement (Table 1 ▸). The 64 kDa enzyme contains an N-terminal Ig-like domain of approximately 80 amino-acid residues and a C-terminal catalytic domain of approximately 465 amino-acid residues (Fig. 1 ▸ and Supplementary Fig. S1). The substrate-binding cleft assumes the shape of an open channel. While all of the secondary-structure elements within the N-terminal portion are β-strands, the enzyme as a whole contains approximately 40% each of α-helices and random coil.

The overall fold of the Ig-like domain largely resembles those of related structures, with some differences found in loop regions. It contains seven β-strands grouped into sets of three (strands 1, 4 and 5) and four (strands 2, 3, 6 and 7) to form two strictly antiparallel β-sheets. The latter sheet is bifurcated owing to Pro74 kinking β-strand 7 into two halves. The N-terminus is located at the end of a cavity near the catalytic domain. In both crystal structures presented here a citrate ion occupies the space where the closest homologs of J30 within GH family 9, including CbhA from *C. thermocellum* (CtCbhA; PDB entry 1ut9), have additional upstream amino acids that contribute to an eighth β-strand at the interface to the catalytic domain (Supplementary Fig. S1; Pereira *et al.*, 2009 ▸; Schubot *et al.*, 2004 ▸; Okano *et al.*, 2015 ▸). The absence of this strand, and the associated hydrogen bonds that would connect it to β-strands 1 and 7 (J30 numbering) as well as to the catalytic domain, may contribute to the lower thermal tolerance of J30. The interface between the Ig-like domain and the catalytic domain is stabilized by hydrophobic interactions, a salt bridge between Asp47 and Lys510, and seven hydrogen bonds between Glu4 and Arg431, Ile8 and Arg447, Asp42 and Asn395, Ala44 and Ala509, Asp47 and Tyr397, and His51 and both Gly396 and Arg398. Their relative arrangement is similar to other GH family 9 members with an Ig-like domain, such as LC-CelG, AaCel9A and CtCel9A (Okano *et al.*, 2015 ▸; Pereira *et al.*, 2009 ▸).

The predominant architectural element of the catalytic domain is an (α/α)_6_-barrel structure composed of 12 α-helices arranged in an alternating pattern as is typical within GH family 9 (Juy *et al.*, 1992 ▸) as well as other families (Parsiegla *et al.*, 1998 ▸). The barrel is built upon an inner ring of six parallel α-helices (2, 6, 8, 11, 13 and 18; arranged clockwise in Fig. 1 ▸) framed by an outer ring of six α-helices of opposite direction (5, 7, 9, 12, 14 and 1). The outer helices are tilted at an angle relative to the inner helices. While some loops between the barrel helices contain extended sections of random coil as well as additional secondary-structure features, the loops between any inner helix and its adjacent outer helix are mostly short (between the pairs 6/7, 8/9, 11/12 and 13/14; Supplementary Fig. S1). As is typical for these (α/α)_6_-barrels, the circle closes with the most C-terminal α-helix 18 neighboring α-helix 1. α-Helix 10 can be considered a continuation of α-helix 9, with the helices being separated only by a kink caused by Phe339. In the catalytic domain, the more extended segments between barrel helices contain five additional α-helices, one 3_10_-helix and six short β-strands forming two separate sheets. Apart from the helices of the (α/α)_6_-barrel, α-helix 17 is the only conserved helix.

The substrate-binding channel extends from a region near the N-terminal ends of the inner barrel helices (corresponding to where the nonreducing end of the bound substrate would be) towards a more unstructured portion of J30 flanked mostly by random coil (Fig. 1 ▸). It runs at an angle relative to the barrel structure, coming closest to secondary-structure elements at the barrel helices 13 and 18 as well as the conserved α-helix 17. Most of the residues expected to directly bind substrate or catalyze the reaction are positioned in random-coil sections of the enzyme, with only Tyr151 (α-helix 2), Arg469 (15), Tyr519 (17) and Tyr528 (18) found on secondary-structure elements (Supplementary Fig. S1). Many of the secondary-structure elements listed above appear to provide a scaffold behind the residues that directly interact with substrates.

### 3.2. The structure of the triple mutant J30 CCH   

**Figure 2:**
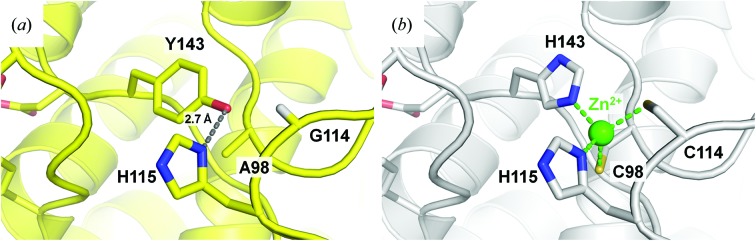
The Zn^2+^-introduction site within the crystal structures of J30 wt and J30 CCH. (*a*) In J30 wt, a hydrogen bond connects the side chains of His115 and Tyr143. Without interfering, the H^α3^ of Gly114 and the side chain of Ala98 point towards them. (*b*) The crystal structure of J30 CCH shows the successful residue mutations and Zn^2+^ incorporation. The metal ion is coordinated by the side chains of Cys98 (2.3 Å distance), Cys114 (2.4 Å), His115 and His143 (both 2.1 Å).

The crystal structure of J30 CCH was solved to a resolution of 1.46 Å. The electron density is consistent with the presence of a Zn^2+^ ion in the predicted site (Fig. 2 ▸ *b*). Metal coordination is mediated by the side chains of Cys98, Cys114, His115 and His143, and effectively replaces one hydrogen bond formed by His115 and Tyr143 in the wild-type enzyme (Fig. 2 ▸ *a*). Among the GH family 9 structures with a homologous Zn^2+^-binding site, CtCel9A, AaCel9A (Pereira *et al.*, 2009 ▸), Cel9M from *Clostridium cellulolyticum* (PDB entry 1ia7; Parsiegla *et al.*, 2002 ▸) and CelT from *C. thermocellum* (PDB entry 2yik; Kesavulu *et al.*, 2012 ▸) share the same mode of chelation, whereas LC-CelG features an aspartate residue at the position equivalent to Cys114 of J30 CCH (Supplementary Fig. S1; Okano *et al.*, 2015 ▸). In general, histidine, acidic and particularly cysteine side chains are the common ligands of structural Zn^2+^ ions (Pace & Weerapana, 2014 ▸). While CtCbhA contains a hydrogen bond between the side chains of a tyrosine and a histidine residue, similar to J30 (Schubot *et al.*, 2004 ▸), some GH family 9 members, including an endoglucanase from *Perinereis brevicirris* (PDB entry 4zg8), have a serine taking the role of the histidine (Arimori *et al.*, 2013 ▸; Sakon *et al.*, 1997 ▸; Mandelman *et al.*, 2003 ▸; Petkun *et al.*, 2015 ▸). Other structures lack the hydrogen bond entirely because a phenylalanine replaces the tyrosine (Khademi *et al.*, 2002 ▸; Brunecky *et al.*, 2013 ▸), or there is a different backbone fold, as is the case in PpGlcNase (Honda *et al.*, 2016 ▸) and the chitobiase from *V. parahaemolyticus*. The Zn^2+^ ion is located at a distance of 14 Å from the center of the active site towards what would correspond to the reducing end of the bound substrate. The root-mean-square deviation between the backbone C, N and C^α^ atoms of the crystal structures of the wild-type and triple-mutant enzymes is 0.094 Å, *i.e.* they are virtually identical. In the crystal structure of J30 CCH we observe a lower average isotropic *B* factor than in the crystal structure of J30 wt (Table 1 ▸). The local atomic displacement parameters of the residues neighboring residues 98, 114 and 143 show a similar trend, suggesting stabilization of the mutated site through the introduction of the Zn^2+^ ion.

### 3.3. The modes of substrate binding and catalysis   

**Figure 3:**
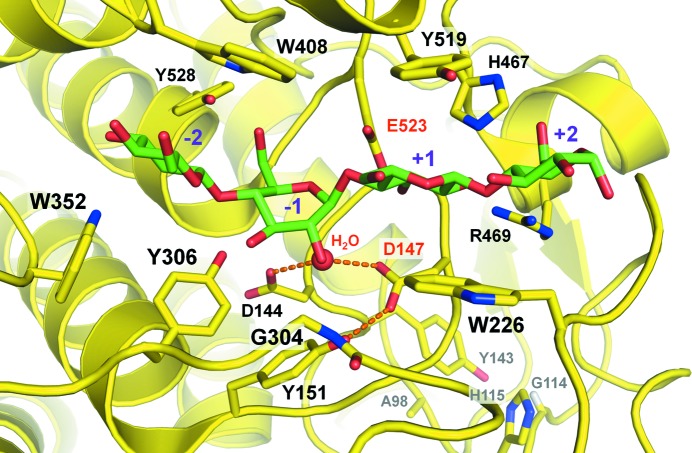
A cellotetraose molecule from the CtCbhA mutant E795Q (PDB entry 1rq5) fitted into the active site of the crystal structure of J30 wt. The individual rings are marked +2 to −2 according to their relative position with respect to the glycosidic bond that would be cleaved by the action of the side chains of Asp147 and Glu523 (labeled in red). Dashes denote hydrogen bonds immediately involved in the nucleophilic attack of the hydrolyzing water molecule (red sphere). The engineered Zn^2+^ site is at the bottom right and is shown with gray labels. The residue-label font sizes reflect the proximity to the viewer.

With the most prominent secondary-structure elements and key residues conserved, the question of the underlying details of substrate binding by J30 is raised. We have superposed the crystal structures of J30 wt and the inactive CtCbhA mutant E795Q, which was co-crystallized with cellotetraose (PDB entry 1rq5; Schubot *et al.*, 2004 ▸), in an effort to model the ligand into the active site of J30 (Fig. 3 ▸). Glu523 of J30 corresponds to Glu795 of CtCbhA (Supplementary Fig. S1).

The highly conserved acidic side chains of Asp147 and Glu523 are directly involved in the enzymatic catalysis. Glu523 acts as a proton donor, while Asp147 deprotonates the depicted water molecule for its nucleophilic attack (Davies & Henrissat, 1995 ▸). This model is in agreement with GH family 9 members following the inverting reaction mechanism with regard to the anomeric C atom of the ring in the −1 position, as Asp147 and Glu523 interact with the substrate from opposite sides. The nucleophilic attack is aided by a network of hydrogen bonds involving the highly conserved Asp144 and the moderately conserved Tyr151. The arrangement is nearly identical to that found in AaCel9A (Pereira *et al.*, 2009 ▸) and is similar to the well described geometry within cellulase E4 from *Thermomonospora fusca* (Sakon *et al.*, 1997 ▸; Fig. 3 ▸).

The carbohydrate ring in the +1 position is stacked between the side chains of Trp226 and Tyr519. All GH9 family members with solved structures contain aromatic residues in these positions. This ring is further held in position through hydrogen bonds involving His467 and Arg469, both of which are highly conserved among the same structures except for the less closely related PpGlcNase and the chitobiase from *V. parahaemolyticus*. The same conservation pattern is found for Tyr306, Trp352 and Trp408 that interact with the rings in the −1 and −2 positions through hydrogen bonds (Tyr306 and Trp352) or ring stacking (Trp408). The side chain of the latter residue is stabilized through a moderately conserved hydrogen bond to Tyr528. The hydrogen bond between the main-chain N atom of Gly304 and the carbo­hydrate moiety in the −1 position is only shared between J30 and its closest homologs LC-CelG, CtCel9A, AaCel9A and CtCbhA.

Figs. 2 ▸ and 3 ▸ together illustrate that the separation of the Zn^2+^-introduction site from the active site is a result of the side chain of His143 pointing in the opposite direction to Asp144 and Asp147. As this orientation of the His143 side chain is similar to that of Tyr143 in J30 wt, a negative impact of metal ion binding on the catalytic activity of the enzyme would not be expected. In contrast, stabilization of the stretch of random coil containing His143, Asp144 and Asp147 could be explained by the triple mutation and introduction of Zn^2+^ through connection of the three loops containing Ala98, Gly114 and His115, and Tyr143 through the metal ion.

### 3.4. The influence of metal introduction on the thermal tolerance of J30   

**Figure 4:**
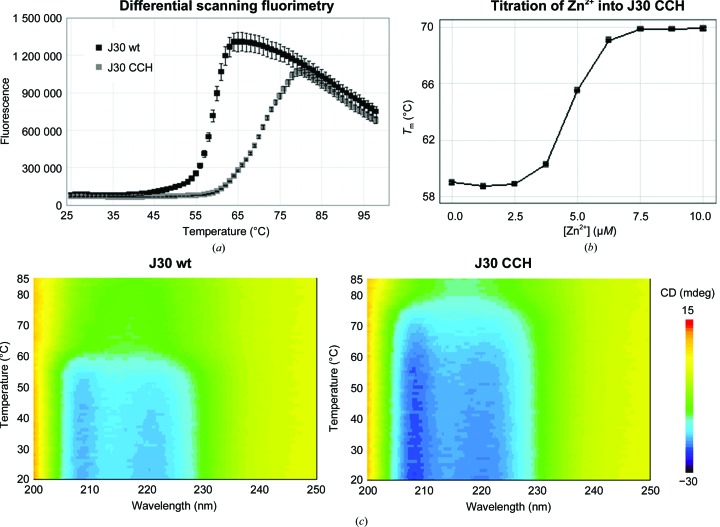
J30 CCH exhibits an increased thermal tolerance compared with the wild-type enzyme and Zn^2+^-stripped J30 CCH. (*a*, *b*) Differential scanning fluorimetry. The standard errors of triplicates are shown. (*a*) The introduction of Zn^2+^ increased the calculated *T*_m_ by 11.2°C. (*b*) The thermal tolerance of J30 CCH after incubation with EDTA equaled that of the wild-type enzyme, but could be regained by the addition of Zn^2+^. (*c*) Circular dichroism. The secondary-structure elements of the triple mutant withstand considerably higher temperatures than those of J30 wt.

In differential scanning fluorimetry assays, the enzyme variants are seen to unfold differently (Fig. 4 ▸ *a*). Based on the inflection points of the curves, the *T*_m_ of J30 CCH (70.1°C) is 11.2°C higher than that of the wild-type enzyme (58.9°C). That this increase is owing to the presence of the Zn^2+^ ion was confirmed by its removal using EDTA, which reduced the *T*_m_ of the triple mutant to the same level as that of J30 wt (Fig. 4 ▸ *b*). Reintroduction of the ion into J30 CCH fully restores the thermal tolerance of the enzyme. CD melting curves support these observations (Fig. 4 ▸ *c*). The local minima at 208 and 222 nm typical of α-helical content are consistent with the structure of the catalytic domain. Their decreases in amplitude with increasing temperatures probably reflect a loss of structural integrity of the catalytic domain. Based on the DSF data, the J30 CCH mutant has a modest 5 µ*M* affinity for Zn^2+^ (Fig. 4 ▸ *b*; Kochańczyk *et al.*, 2015 ▸). However, incorporation of the Zn^2+^ ion still stabilizes the protein and contributes to the difference in thermal tolerance between the wild-type and mutant enzymes.

### 3.5. Enzymatic specificity of J30 wt and J30 CCH   

**Figure 5:**
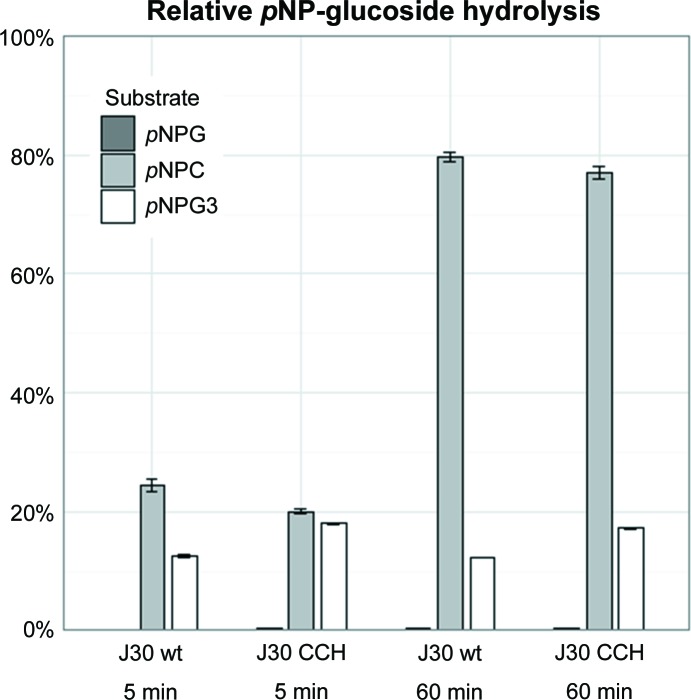
Enzyme substrate comparison. J30 wt and J30 CCH follow a distinct activity pattern on selected glucoside derivatives. The absorbance of *p*NP reflects the hydrolysis of its β-glucosides with one (*p*NPG), two (*p*NPC) or three (*p*NPG3) glucose moieties. 100% refers to positive controls using 3.6 *M* NaOH.

J30 has been reported to hydrolyze the cellotriose analog *p*NPC but not the cellobiose analog *p*NPG (Gladden *et al.*, 2014 ▸), and previous enzymatic studies have revealed the substrate specificity of AaCel9A (Eckert *et al.*, 2002 ▸, 2009 ▸). We examined the substrate specificities of J30 wt and J30 CCH towards 4-nitrophenyl glucosides containing one, two or three sugar moieties, and compared the release of free *p*-nitrophenolate (*p*NP) with substrate hydrolysis by 3.6 *M* NaOH (representing 100%; Fig. 5 ▸). Both variants had the same activity profiles across the three tested substrates, confirming the previous results, and are similar to AaCel9A: they are unable to hydrolyze *p*NPG and produce more detectable *p*NP from *p*NPC than from the cellotetraose analog *p*NPG3. While the triple mutant produced slightly more *p*NP from *p*NPG3 than the wild-type enzyme at 50°C, both ultimately appear to favor the hydrolysis of *p*NPG3 to cellobiose and noncleavable *p*NPG over the production of cellotriose and *p*NP, thereby paralleling the experimental data for AaCel9A (Eckert *et al.*, 2002 ▸).

### 3.6. Catalytic profiling of J30 wt and J30 CCH   

**Figure 6:**
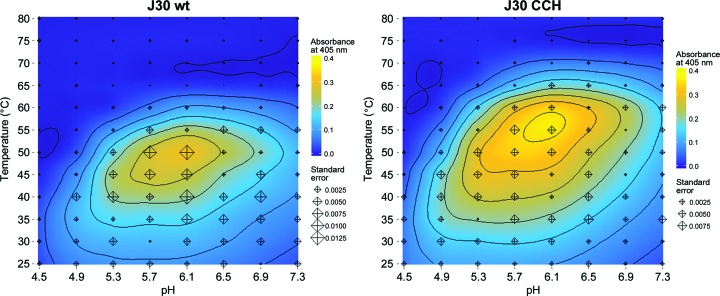
Enzymatic assays. The increase in thermal tolerance is paralleled by a shift in the catalytic profiles of J30 wt and J30 CCH. The *p*NPC turnover of both enzyme variants is plotted dependent on reaction temperature and pH, showing an increase in the optimal temperature (contour interval of 0.05). Each data-point triplicate is represented by a diamond, the size of which corresponds to its standard error.

The rate of product formation in enzymatic catalysis, and in a hydrolysis reaction in particular, is inherently dependent on the temperature and pH. The nature of these relationships in the case of J30 wt and J30 CCH was studied using *p*NPC as a substrate (Fig. 6 ▸). Both enzyme variants show a catalytic optimum around pH 6, which is consistent with the reaction mechanism. The incorporation of the Zn^2+^ ion increases the temperature optimum of J30 for *p*NPC turnover by 5–10°C, and its influence on catalysis is merely physical and not chemical. These findings are confirmed by the DSF and CD data, and are consistent with prior characterization of the wild-type J30 enzyme (Gladden *et al.*, 2014 ▸).

## 4. Conclusions   

The positive effect of Ca^2+^ binding on the thermal tolerance of the cellulase CtCel9A has been shown previously, as has the correlation of a Zn^2+^-binding site with enzyme activity and structural integrity (Chauvaux *et al.*, 1990 ▸, 1995 ▸). Our design of a mutant version of J30 based on structural data further underlines this relationship. Here, the majority of the increase in stability is likely to be a result of the net exchange of a hydrogen bond between the side chains of His115 and Tyr143 (J30 wt) for four coordinating bonds between the Zn^2+^ ion and the side chains of Cys98, Cys114, His115 and His143 (J30 CCH; Fig. 2 ▸). This stabilization may in part result from three separate sections of the polypeptide chain interacting *via* the Zn^2+^ ion. The structural and biochemical data strongly suggest that the metal-binding site is purely structural, yet it is also proximal to the active-site residues Asp144 and Asp147 and the region of the substrate cleft where the reducing end binds.

In contrast to its closest homologs, J30 does not contain bound Ca^2+^ ions. Three Ca^2+^-binding sites are found in CtCel9A, two of which, in varying combinations, are found in AaCel9A, CtCbhA and LC-CelG. Two of the Ca^2+^ sites are found in loop regions with backbone geometries different to those in J30, corresponding to J30 residues 175–198 and 471–475 (Juy *et al.*, 1992 ▸). The third site is located in a region of conserved backbone fold, and is found in the crystal structures of all four homologs; it is referred to as ‘B’ in Chauvaux *et al.* (1995 ▸). In J30, the side-chain amino group of Lys308 forms hydrogen bonds to the Gly353 O atom and the Glu313 and Glu314 carboxyl groups at precisely the same site. Hence, the introduction of a Ca^2+^ ion into J30 by point mutations in this region may work in a similar manner to the Zn^2+^ example, but would be likely to require loop remodeling in the case of the other two sites.

Unlike the incorporation of disulfide bonds to cross-link otherwise more loosely connected sections of a polypeptide chain, our approach is not limited to secreted proteins. We have taken advantage of a Zn^2+^-binding site seen in homologs of the target enzyme, and as a result have improved the thermal stability and the temperature optimum of the enzyme. This strategy is likely to be proven to be promising in the future in a more general context: the *de novo* introduction of metal ion binding into proteins. Such an approach is undoubtedly more challenging when there are no metal ion-binding homologs that can be used as a reference. In particular, recent efforts show how interaction networks beyond the geometry of the immediate site to be designed need to be considered (Guffy *et al.*, 2016 ▸). However, computational approaches for protein redesign have improved considerably in the last decade (Simons *et al.*, 1999 ▸; Huang *et al.*, 2016 ▸), and could enable the creation of thermally and chemically more robust proteins that are useful for medical and biotechnological applications. The increases in both the thermal tolerance and the optimal catalytic temperature of J30 address bottlenecks in biomass deconstruction. In this step of biofuel production, the robustness of the enzymes translates directly into the cost of the final product (Blanch *et al.*, 2011 ▸).

## Supplementary Material

PDB reference: wild-type J30, 5u0h

PDB reference: J30 CCH, 5u2o

Supplementary Figure S1.. DOI: 10.1107/S2059798318006678/yt5103sup1.pdf

## Figures and Tables

**Table 1:** Data-collection, refinement and model statistics Values in parentheses are for the outer shell.

	J30 wt	J30 CCH
PDB code	5u0h	5u2o
Data-collection statistics		
Beamline	7-1, SSRL	5.0.1, ALS
Wavelength (Å)	1.1271	0.9774
Crystal-to-detector distance (mm)	200	180
φ collected/Δφ (°)	120/1	180/1
Exposure time (s)	3	1
Space group	*P*6_5_22	*P*6_5_22
Unit-cell parameters		
*a*, *b*, *c* (Å)	91.06, 91.06, 316.66	90.96, 90.96, 316.32
α, β, γ (°)	90, 90, 120	90, 90, 120
Resolution (Å)	76.52–1.70 (1.74–1.70)	70.51–1.46 (1.50–1.46)
Measured reflections	1220921 (79778)	2888558 (210630)
Unique reflections	86505 (6276)	134366 (9693)
Completeness (%)	100.0 (100.0)	99.8 (99.2)
Multiplicity	14.1 (12.7)	21.5 (21.7)
*R*_merge_ † (%)	13.5 (131.3)	13.8 (170.4)
〈*I*/*σ*(*I*)〉	19.3 (2.2)	17.8 (2.3)
CC_1/2_ (%)	99.9 (70.6)	99.9 (71.5)
Wilson *B* factor (Å^2^)	15.9	13.4
Refinement and model statistics		
Resolution (Å)	55.9–1.70 (1.74–1.70)	70.51–1.46 (1.50–1.46)
*R*_cryst_ ‡ (%)	14.09 (23.39)	13.97 (22.81)
*R*_free_ § (%)	16.57 (25.51)	15.12 (24.98)
No. of molecules		
Protein	1	1
Water	765	800
Citrate	1	1
Glycerol	3	4
Zn^2+^	0	1
No. of protein residues	543	543
R.m.s.d.¶		
Bond lengths (Å)	0.008	0.007
Bond angles (°)	0.981	0.924
Clashscore	0.23	0.57
Average isotropic *B* factor (Å^2^)		
Overall	20.6	19.4
Macromolecules	18.0	16.6
Solvent	34.8	34.0
Ligands	32.3	30.4
Ramachandran plot		
Favored region (%)	98	98
Allowed region (%)	1.6	1.6
Outliers (%)	0.18	0.18
Rotamer outliers (%)	0.67	0.67

† *R*_merge_ = 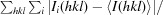 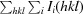, where *I*_*i*_(*hkl*) is the intensity of an individual measurement of the reflection and 〈*I*(*hkl*)〉 is the mean intensity of the reflection.

‡ *R*_cryst_ = 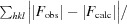 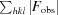, where *F*_obs_ and *F*_calc_ are the observed and calculated structure-factor amplitudes, respectively.

§ *R*_free_ was calculated as *R*_cryst_ using 2000 randomly selected reflections that were omitted from the structure refinement.

¶ The root-mean-square deviations of bond angles and lengths were calculated based on the conformation-dependent library (Moriarty *et al.*, 2014 ▸, 2016 ▸).
