# Supplementary material for: Engineering glycoside hydrolase stability by the introduction of zinc binding
Source: Acta Crystallogr D Struct Biol. 2018 Jun 27;74(Pt 7):702–10. doi: 10.1107/S2059798318006678 (PMC6038386; doi:10.1107/S2059798318006678)
Supplement: Supplementary file 1 [file d-74-00702-sup1.pdf]

STRUCTURAL
BIOLOGY

Volume 74 (2018)

Supporting information for article:

Engineering glycoside hydrolase stability by the introduction of zinc binding

Thomas L. Ellinghaus, Jose H. Pereira, Ryan P. McAndrew, Ditte H. Welner, Andy M. DeGiovanni, Joel M. Guenther, Huu M. Tran, Taya Feldman, Blake A. Simmons, Kenneth L. Sale and Paul D. Adams

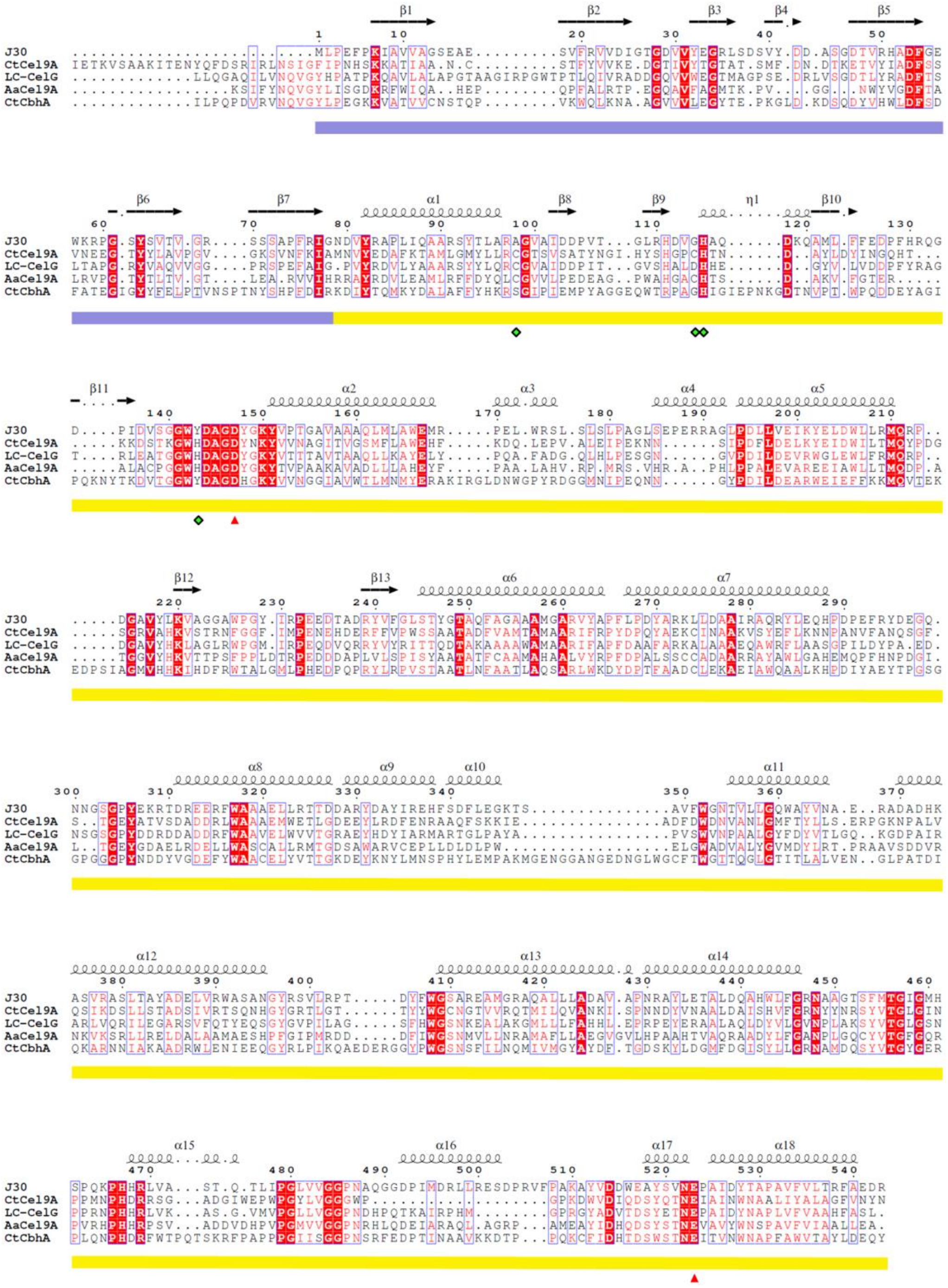

Figure S1 Sequence alignment based on the crystal structures of J30 and its closest homologs. The amino-acid numbering, domain color coding (same as in Figure 1) and secondary-structure elements refer to J30 wt (α: *α*-helices; β: *β*-strands; η: 3_{10}-helix). The covered residues comprise those present in the crystal structures of CtCel9A (amino acids 35-575), LC-CelG (30-577), AaCel9A (7-534) and CtCbhA (208-815). Green and black diamonds denote the locations of the Zn^{2+}-coordinating residues in the triple mutant J30 CCH, and red triangles point towards the catalytic residues. The bipartite *β*-strand 7 (residues 70-72 and 75-77) is depicted as a continuous strand for the sake of simplicity.
